# Fifteen research needs for understanding climate change impacts on ecosystems and society in the Norwegian High North

**DOI:** 10.1007/s13280-023-01882-9

**Published:** 2023-06-07

**Authors:** Zina Kebir, Catherine Chambers, André Frainier, Vera Hausner, Ann Eileen Lennert, Jennifer Lento, Amanda Poste, Virve Ravolainen, Angelika H. H. Renner, David N. Thomas, Kerry Waylen

**Affiliations:** 1 Department of Arctic and Marine Biology, The Arctic University of Norway (UiT), Biologibygget, Framstredet 39, 9019 Tromsø, Norway; 2 Stefansson Arctic Institute and Research Manager at University Centre of the Westfjords, Suðurgata 12, 400 Ísafjörður, Iceland; 3 Norwegian Institute for Nature Research (NINA), FRAM – High North Research Centre for Climate and the Environment, Hjalmar Johansens Gate 14, Tromsø, Norway; 4 Department of Biology and Canadian Rivers Institute, University of New Brunswick, 10 Bailey Drive, Fredericton, NB E3B 5A3 Canada; 5 Norwegian Polar Institute, FRAM – High North Research Centre for Climate and the Environment, Hjalmar Johansens Gate 14, Tromsø, Norway; 6 Institute of Marine Research, FRAM – High North Research Centre for Climate and the Environment, Hjalmar Johansens Gate 14, Tromsø, Norway; 7 Faculty of Biological & Environmental Sciences, Helsinki Institute of Sustainability Science (HELSUS), University of Helsinki, Yliopistonkatu 3, 00014 Helsinki, Finland; 8 Social, Economic and Geographical Sciences Department, James Hutton Institute, Cragiebuckler, Aberdeen, AB15 8QH Scotland, UK

**Keywords:** Climate change, Cross-ecosystem impacts, Horizon scan, Knowledge gaps, Priority setting, Socioecological system

## Abstract

There is an urgent need to understand and address the risks associated with a warming climate for ecosystems and societies in the Arctic and sub-Arctic regions. There are major gaps in our understanding of the complex effects of climate change—including extreme events, cascading impacts across ecosystems, and the underlying socioecological dynamics and feedbacks—all of which need collaborative efforts to be resolved. Here, we present results where climate scientists, ecologists, social scientists, and practitioners were asked to identify the most urgent research needs for understanding climate change impacts and to identify the actions for reducing future risks in catchment areas in the Norwegian High North, a region that encompasses both Arctic and sub-Arctic climates in northern Norway. From a list of 77 questions, our panel of 19 scientists and practitioners identified 15 research needs that should be urgently addressed. We particularly urge researchers to investigate cross-ecosystem impacts and the socioecological feedbacks that could amplify or reduce risks for society.

**Supplementary Information:**

The online version contains supplementary material available at 10.1007/s13280-023-01882-9.

## Introduction

Current and predicted changes in climate are particularly significant for Arctic and sub-Arctic regions, which are experiencing the fastest warming on the planet, two times faster than the global average (IPCC 2019). There, an increase in mean air temperature is expected to induce changes in precipitation and runoff patterns and increase the frequency of extreme climatic events (IPCC 2018, 2021). Climate change impacts terrestrial, freshwater, coastal, and marine ecosystems simultaneously, and by crossing over the boundaries of one ecosystem, climate effects may modify or enhance effects on adjacent ecosystems (cross-ecosystem impacts) (Loreau et al. 2003; Gounand et al. 2018). For example, in many Arctic and sub-Arctic regions, changes affecting snow (snow accumulation, thawing events) are impacting the timing and magnitude of spring snowmelt floods that play an important role in the transport of particulate matter (terrestrial carbon, nutrients, contaminants) to downstream freshwater and coastal ecosystems (Kane et al. 2003; Finlay et al. 2006). Such cross-ecosystem impacts have implications for Indigenous peoples and local communities and for industries such as tourism, aquaculture, fisheries, hydroelectric power plants, reindeer husbandry, and agriculture (Gounand et al. 2018). Understanding the socioecological impacts of such broad-scale changes requires a collaborative approach that draws on both research and practice, including the knowledge that Indigenous peoples and local communities have accumulated about the interactions between climate, ecosystems, and society. Early identification of plausible future risks for management of ecosystems and adaptation of communities could reduce the probability of sudden unexpected confrontation with major climate, environmental, and societal changes (Sutherland et al. 2009).

The Norwegian High North (including Svalbard) encompasses both Arctic and sub-Arctic climates and provides a suitable case study region for studying these topics, as, currently, little is known about the complexity of climate change impacts in Arctic and sub-Arctic catchments and the implications a warming climate has for the multiple ecosystems and societies involved. In the face of this complexity and uncertainty, a mixture of expertise and perspectives is required to identify the priorities for research and action (Armitage et al. 2011).

In this study, we assembled a panel of climate scientists, ecologists, social scientists, and practitioners to identify and define the urgent research challenges and knowledge gaps that need to be filled to better understand these cross-ecosystems and societal impacts. We used horizon scanning which is a research method with the goal to identify, describe and examine potential medium to long-term phenomena (threats, mitigations, and solutions) that are not well recognized within a certain field (Sutherland et al. 2014). Other methods such as literature reviews can lead to similar results but typically do not involve collaborative and transdisciplinary work. Here the goal was to gather actors from multiple sectors to increase the chances of not omitting any possible issues. Our horizon scan encouraged researchers to work with policy makers and practitioners to identify climate change-induced impacts.

More specifically, we focused on how we could advance our understanding of cross-ecosystem impacts, socioecological dynamics, and adaptation actions by focusing on physical changes in catchment areas of the Norwegian High North. Despite the importance of understanding the linkages that exist between terrestrial, freshwater, and marine ecosystems and the impacts that climate change has on their functions and services, these ecosystems are often treated separately in research and management plans in Norway. Moreover, climate change adaptations related to the important linkages between aquatic and terrestrial domains and the communities around them are not yet an integrated part of management strategies in the circumpolar region. Catchments in the Norwegian High North support many different sectors such as fishery, agriculture, and research and are also a major intersection between terrestrial, freshwater, and marine ecosystems. By taking catchment-scale approaches, it is possible to capture cross-ecosystems interactions and cover broad climate, ecological, and social contexts. Here, our goal was to co-define priorities for research needs related to cross-ecosystem linkages and feedbacks and to gain insights into the socioecological linkages that can be impacted by climate change in the Norwegian High North.

## Identification of research needs

**Fig. 1:**
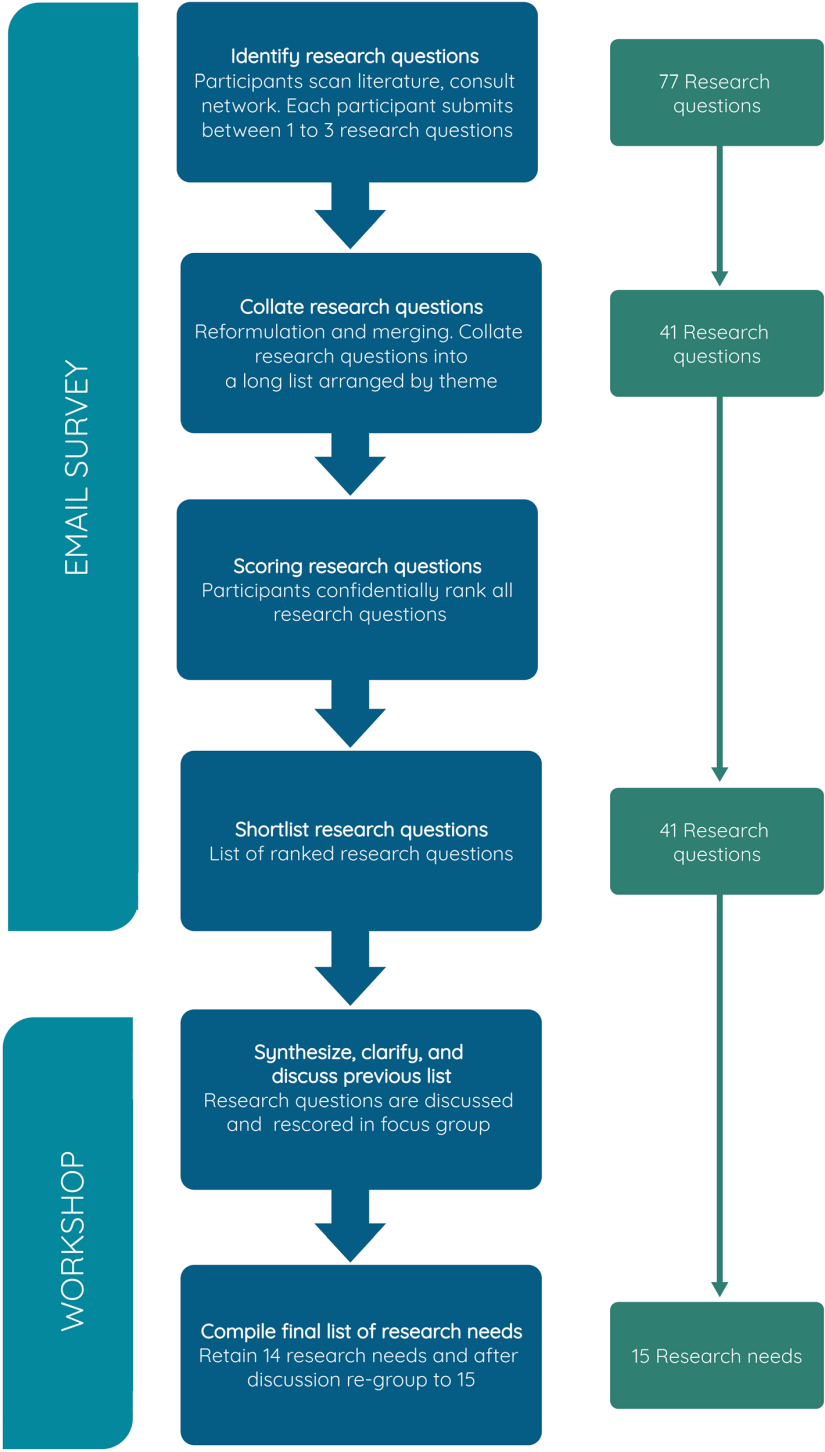
Process for identifying and evaluating research needs for the horizon scan

For the horizon scan, we used a modification of the Delphi technique (Sutherland et al. 2014) which was first developed as a forecasting tool. The process is inclusive, transparent, and encourages communication between experts from varied backgrounds (Rowe and Wright 1999; Sutherland et al. 2014). A broad-scale survey allowed us to gather experts’ opinions about research gaps and research needs (Fig. 1).

In March 2022, we selected experts through snowballing by asking research participants to assist in identifying potential subjects. We started the selection by approaching the large research network from the FRAM—High North Research Centre for Climate and the Environment (Fram Centre) while also scanning the web for potentially relevant participants. A total of 193 participants were contacted to contribute to the horizon scan. About half of these were Norwegian researchers from the 20 environmental institutes hosted by the High North Centre for Climate and the Environment, wherein only 13% were social scientists. We also invited 80 non-academic participants distributed among industry, public and NGOs. In addition, 16 international experts were invited. The potential participants were requested via email to respond to an online survey and to submit information about one to three perceived research questions. The point of this survey was to generate a broad and diverse range of research questions that the workshop participants could use as a basis for ranking research needs. Unfortunately, and despite our efforts to stimulate participation in the survey by use of weekly reminders, only 14% responded. Nonetheless, we received a total of 77 research questions suggested by the participants.

The research questions were thoroughly examined to identify the key themes and eliminate redundancy. In some cases, two or more issues appeared to be similar and were therefore merged and reformulated for the next stage. This process resulted in a final list of 41 research questions (see Appendix S1). These were classified in four themes (biodiversity and food web; climate-land–water: fluxes; ecosystems and society: climate adaptation; and ecosystems and society: management) to simplify the procedure and encourage participation from all potential respondents. The potential 193 participants were contacted via email again to participate to the scoring phase. The participation rate was slightly higher in the scoring phase than in the identification phase, with 18% of participants responded. Participants independently and confidentially scored each of the 41 research questions from 1 (low importance, definitely discard) to 10 (high importance, definitely retained). Participants were also offered an opportunity to suggest one additional research question per theme. Each phase of the survey lasted 2 weeks and the scoring phase ended mid-April.

Parallel to the online survey, a panel of experts was selected and invited to participate in a hybrid workshop (virtual and in person) at the end of April 2022. Experts were divided into groups according to their field of expertise and the themes used in the survey. Each group was composed of 4–6 participants including 1 to 2 international experts and all the groups except of the group of the climate-land–water: fluxes theme (due to last minute cancelation) had 2 non-academic representatives. We had, among others, representants from the reindeer herders, fish and game management or managers at the county level—all actively working in Northern Norway. A group leader was assigned to each group and was responsible for delivering the resulting list of the scored research questions to the member of its group ahead of the workshop. The workshop was divided into two sessions: a smaller focus group session and a plenary session. The focus group session allowed experts to discuss in depth the research questions corresponding to the theme they were assigned to (biodiversity and food web; climate-land–water: fluxes; ecosystems and society: climate adaptation; and ecosystems and society: management). The goal was to merge and/or reformulate the research questions into a final list of 3 to 5 research needs per group (see Appendix S2). Despite the digital participation of some experts and the time zone differences, the discussion was rich and detailed. During the plenary session, each group leader presented the results from the focus group session and briefly explained the process that led to the 3 to 5 research needs. Next, each panel member had a few minutes to express his/her point of view on the overall list of research needs.

**Fig. 2:**
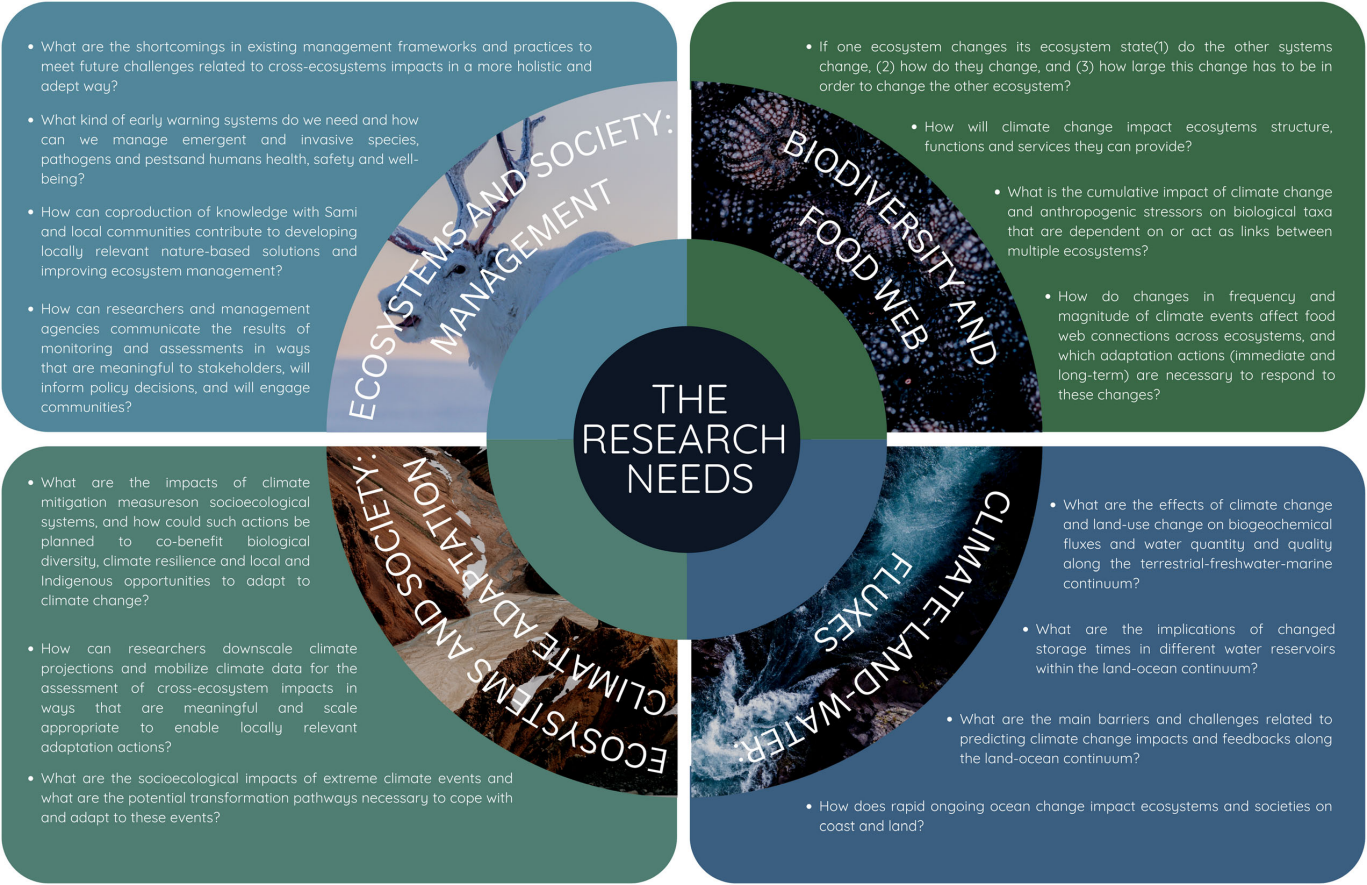
The 15 research needs identified during the horizon scan

A stimulating discussion followed, leading to the modification of the list until consensus was reached. At the end, 14 research needs were identified. During the writing and editing, as further research, the co-authors identified one research question that had been merged with another research question but was important to treat on its own and decided to add this issue to the final list. In addition, the group moved one question in climate adaptation to the management section as it was agreed that it seemed more relevant there. The final 15 identified research needs are listed in Fig. 2. They are presented in italic text and discussed in their respective thematic groups below (B: biodiversity and food web; F: climate-land–water: fluxes; C: ecosystems and society: climate adaptation; and M: ecosystems and society: management). The research questions (Q’s, listed in Appendix S1 and Table S1) leading up to those 15 final research needs are also mentioned.

## The research needs

### Biodiversity and food web

The observed impacts of climate change on biodiversity and ecosystems is well documented for the Arctic and sub-Arctic regions (CAFF 2013; IPCC 2018). As temperatures rise, thawing permafrost releases large amounts of carbon to the freshwater and coastal systems and to the atmosphere (Tank et al. 2020; Karlsson et al. 2021). Rising temperatures also affect the duration and characteristics of the winter and summer seasons, reducing thermal insulation due to reduced land snow-cover, altering lake temperature and light regimes due to reduced lake ice-cover, and allowing new animals, plants, parasites, and microorganisms to establish in areas previously unsuitable for them (Vowles and Björk 2019; Calizza et al. 2022). Although some climate change effects on biodiversity and food webs are well understood in the Norwegian High North, most of this knowledge is restricted to within pre-defined boundaries, as in lakes or in the tundra vegetation. To date, there is not a good understanding of how changes in one ecosystem may affect the other, nor how those changes may escalate from one ecosystem to the other and cause other currently unexpected effects on ecosystems and human societies (Macdonald et al. 2015).

Concerns about the response of multiple ecosystems to climate change were expressed in terms of understanding ecosystems resilience (Q1.1), consequences for local (Q1.4–1.5) and migratory (Q1.3) species. In particular, participants expressed concerns about the future conditions of suitable habitats for reindeer grazing (Q1.7), hunting, berry picking, and fishing (Q1.5), changes in forest vegetation (Q1.4), and availability of marine resources (Q1.8). Some participants also expressed more general issues to understand the environmental factors driving changes in biodiversity and food webs (Q1.2,1.5,1.8), and more methodological questions, as in how to compare food webs across ecosystems (Q1.6) and possibilities for using new methods such as eDNA for monitoring across all diversity levels (Q1.9). The identified research needs are described in the subsections below.

*B1: If one ecosystem changes its ecosystem state (e.g., from kelp-dominated to urchin-dominated coastal zone, from tundra to birch forest ecosystem, from benthic-dominated to pelagic-dominated aquatic system or vice-versa) (1) do the other systems change, (2) how do they change, and (3) how large does this change have to be in order to change the other ecosystems?*

One long-held concept in ecology is the idea that ecosystems may change some key characteristics (their ‘state’) nearly permanently due to the effects of external drivers, while this change is hindered by the internal features that promote system stability (Dakos and Hastings 2013), making the system more resilient. Stability can be measured as the amount of change that a system can withstand before it moves to a new state, and once the changes are large enough, the ecosystem changes its fundamental state to an alternative state. For example, kelp-dominated coastal zones can be affected by storms and species invasion and maintain their kelp-rich status, but if the abundance of urchins is sufficiently high, for example, due to reduced abundance of key predators, those ecosystems may become permanently barren (Norderhaug and Christie 2009). Changing the ecosystem state also means changing the composition of species occurring in that area, with large consequences for ecosystem functioning. There may be implications also for other ecosystems when one such ecosystem changes its state. For example, when ecosystems change from tree-dominated to grass-dominated (due to forestry, agriculture, or husbandry), there is a change in nutrients being exported to rivers and lakes, and a change in physical, chemical, and ecological characteristics in the freshwater systems (Frainer and Mckie 2021). This change in nutrient export to freshwater can cause further change in state, making macrophyte-dominated lakes become phytoplankton-dominated lakes due to excess nutrients in the water column, with consequences for carbon burial and export (Brothers et al. 2013). The frequency and extent to which these changes happen in nature are not yet well understood but may play a key role for adaptation and management plans if one ecosystem, with its own limits and management plans, is to be affected by changes in another ecosystem. These concerns were expressed particularly regarding the status of vegetation suitable for reindeer grazing (Q1.7), possibilities for hunting, fishing, and berry picking (Q1.5), and change in ecosystem functions due to the addition of new species following climate change.

*B2: How will climate change impact ecosystems structure (e.g., biodiversity, vegetation strata), functions (flux of nutrients), and services they can provide (e.g., grazing, livestock, berries, game and fish, tourism, cultural, flood protection, aquaculture)?*

Climate change has large and serious impacts for all the ecosystems in the Arctic, changing both their structure, function, and measurable services they provide to humans. Current temperature warming is changing vegetation structure, promoting growth of shrubs in the tundra (greening of the Arctic), advancing the tree line, and allowing grass to advance over areas previously characterized by permafrost (Hope et al. 2015). Permafrost thawing will also cause a higher export of carbon to freshwater and coastal systems (Tank et al. 2020). Animals and plants that are not currently present in the Arctic will be able to settle in this region, which will likely disrupt current food web patterns and interactions with humans. Invasive species are likely to further change ecosystems in unpredictable ways (Wasowicz et al. 2020; Chen et al. 2011). Participants expressed concerns directly related to human use of nature (cloud berry picking, reindeer husbandry, hunting, fishing) (Q1.5,1.7,1.8), invasive species (Q1.4) but also at ecosystem properties, ecosystem functioning, and food webs (Q1.1,1.2,1.4,1.6).

*B3: What is the cumulative impact of climate change and anthropogenic stressors on biological taxa that are dependent on or act as links between multiple ecosystems?*

Climate change causes an increase in the mean temperature and an increase in extreme events, such as heatwaves and storms (IPCC 2018). In the Norwegian High North, marine heatwaves can have severe impacts on coastal communities, and terrestrial heatwaves may also cause disruption to terrestrial ecosystems (ACIA 2004). Changes in precipitation are also expected to result from climate change, including a shift toward higher rainfall in winter (Bintanja and Andry 2017) and more frequent and extreme rain events (Dou et al. 2022). All these changes due to global warming will have short and long-term consequences for Arctic and sub-Arctic ecosystems, and thus affect dependencies and links across multiple ecosystems in the region. In addition, other stressors brought about by modern industrial societies such as intensive forestry, modern agriculture, roads, wind turbines, installation of industries, and mining, among others, add to the stressors brought about by climate change. The understanding of how multiple stressors can affect species biodiversity and ecosystem functioning is an active area of research (Simmons et al. 2021), but little is currently known about how multiple and cumulative stressors affect the Arctic; a field of research that is very methodologically challenging. This concern was expressed by the participants regarding impacts of ocean acidification (Q1.8), species invasion (Q1.4), temperature increase (Q1.5), permafrost thawing (Q1.5), and landscape change (Q1.7).

*B4: How do changes in frequency and magnitude of climate events (extreme events including abrupt increase/decrease in precipitation, temperature, and flood) affect food web connections across ecosystems (e.g., from sea to land, from freshwater to land, from land to sea), and which adaptation actions (immediate and long-term) are necessary to respond to these changes?*

Changes in frequency and magnitude, as well as timing, of climate events can cause large disturbances to connections among species within food webs and ecosystems. Because ecosystems are connected by transport and flux of nutrients across ecosystem boundaries (Loreau et al. 2003), changes in climate that affect one ecosystem are likely to have effects on other ecosystems as well. For example, shorter winter seasons may cause a mismatch in timing for plants, pollinators, and animals who depend on those plants for food, causing disturbances in the food webs across terrestrial, freshwater, and marine ecosystems. Among the participants, connections across ecosystems were relevant for comparing food webs (Q1.6), for improving monitoring activities using eDNA (Q1.9), but also to understand trickle down effects of climate change on nature’s food provisions (Q1.5, 1.7, 1.8).

### Climate-land–water: Fluxes

Climate change impacts on temperature, glacial melt, permafrost thaw, and vegetation change (including documented ‘greening’ of the tundra or insect-mediated defoliation events) alongside land-use changes (e.g., in grazing pressure) are reshaping northern landscapes (AMAP 2021). These changes are occurring alongside climate-driven changes in precipitation and runoff patterns, with many Arctic regions experiencing increased total runoff, changes in seasonality of runoff (including altered timing and magnitude of spring snowmelt floods and increase in autumn and winter runoff) as well as increased frequency of extreme events, such as droughts and floods (IPCC 2021). These changes in terrestrial and freshwater systems are exported downstream, where they meet marine ecosystems that are also under pressure from climate change, e.g., through increased temperatures, changing ocean currents, ocean acidification, and loss of sea ice (AMAP 2021).

Survey respondents highlighted research needs that can be broadly gathered into three overarching and closely related themes: (1) understanding impacts of climate change on the hydrologic cycle and cryosphere, (2) understanding impacts of climate and land-cover changes on biogeochemical cycling of carbon and nutrients, and (3) improved tools for understanding linkages between atmospheric, terrestrial, freshwater, and marine systems.

Research questions focusing on climate change impacts on the hydrologic cycle were related to impacts on runoff quantity and timing (e.g., changing seasonality and flood risk) (Q2.1, 2.6) as well as knowledge gaps related to groundwater-related ecosystems (Q2.9). Identified research needs focusing on impacts of a changing cryosphere included ecological consequences of permafrost thaw (Q2.3) and how changing snow conditions are likely to impact avalanche risk and efficacy of risk mitigation measures (Q2.8). Several questions were also raised related to impacts of climate and land-use change on water quality, including impacts on downstream transport of sediments (Q2.6), nutrients (Q2.1, 2.2, 2.7), carbon (Q2.2, 2.3, 2.5, 2.7) and pollutants (Q2.1) from land to freshwater and coastal systems. Additional research questions focused more directly on knowledge needs related to carbon cycling in northern ecosystems in relation to insect outbreaks (Q2.5), grazing pressure (Q2.10), and potential positive climate change feedbacks related to microbial greenhouse gas production (Q2.7). Finally, several research questions pointed to a need for integrated approaches for understanding land-freshwater-coast (and atmosphere) interactions, both in terms of process understanding related to hydrologic connectivity and biogeochemical cycling (Q2.2, 2.5) as well as through improved integrated modeling tools for coupling terrestrial and aquatic processes (Q2.4). The research needs outlined below were designed to capture and integrate the key themes raised by the survey respondents in a cross-ecosystem context, with one additional question (F4) included to capture a critical knowledge gap that was identified during workshop discussions.

*F1: What are the effects of climate change (including changes in seasonality and extreme events) and land-use change on biogeochemical fluxes and water quantity and quality along the terrestrial-freshwater-marine continuum?*

Changes at catchment-scale are resulting in a broad range of impacts on the mobilization, cycling, and fate of sediments, organic matter, nutrients and contaminants along the continuum from land to sea, via streams, lakes, rivers and coastal waters (Gibson et al. 2022). In particular, inputs of terrestrial particulate and dissolved material can have a broad range of consequences for water quality as well as the structure and function of downstream freshwater and coastal ecosystems (Irrgang et al. 2022). At the same time, fjord and coastal system are under pressure from changes in the physical environment, including changes in hydrography, wind regimes and thus circulation and residence times, which, alongside changes in magnitude and timing of terrestrial runoff, can impact the marine ecosystem and lead to, e.g., shifts in species distributions, coastal darkening or decreased oxygen concentrations (Aksnes et al. 2019; McGovern et al. 2019; Bianchi et al. 2020).

Despite this, the magnitude, timing, and geochemistry of fluxes across ecosystem boundaries (e.g., terrestrial-aquatic and freshwater-marine interfaces) are often poorly characterized, and even less is known about the processing and fate of terrestrial material along the aquatic continuum from headwaters to the ocean. As outlined above, survey participants prioritized several research questions related to climate change impacts on runoff quantity and timing (Q2.1, 2.6), water quality (Q2.1, 2.2, 2.5–2.7), and carbon cycling (including potential climate feedbacks) in northern ecosystems (Q2.3, 2.5, 2.7, 2.10).

*F2: What are the implications of changed storage times in different water reservoirs (e.g., groundwater, surface water, snow, glaciers, fjords) along the land–ocean continuum?*

Broad-scale ‘intensification’ of the Arctic hydrologic cycle, with increases in evaporation rates, precipitation, runoff and melting of glacial and sea ice, can be expected to lead to changes in the distribution, storage and cycling of water (as vapor, liquid, and ice) in the northern environment, both terrestrial and marine (Carmack et al. 2016; Wrona et al. 2016). Changes in snow accumulation, such as winter thaw events, can play a key role in shaping terrestrial vegetation and soil processes, as well as in determining the magnitude of spring snowmelt flooding (Wrona et al. 2016). Changing runoff, including from melting glaciers, plays a key role in mobilization and transport of terrestrial material to the aquatic environment (Gibson et al. 2022), while changing surface water residence time (including residence time of freshwater and terrestrial material in fjords and coastal environments) can play a key role in controlling the processing, biological uptake and fate of, e.g., organic matter and nutrients along the aquatic continuum (Bauer et al. 2013; Frey et al. 2016).

One particular knowledge gap highlighted as a research priority by the study participants was the lack of knowledge related to groundwater-impacted systems (Q2.9). Groundwater in Arctic and sub-Arctic systems is particularly poorly understood, with limited data available regarding groundwater quantity, distribution, geochemistry, and contributions to river flow (Lecher 2017). Recent studies have indicated that groundwater may represent a significant source of organic matter and nutrients to the coastal Arctic Ocean; however, these same studies point to a strong lack of data related to groundwater geochemistry and fluxes (e.g., Connolly et al. 2020). Furthermore, groundwater is typically characterized by distinct (and often more stable) chemistry and temperature compared to surface waters, which can give rise to novel and diverse ecosystems, which are currently poorly characterized and understood (e.g., Huryn et al. 2021).

Although this research question has links to questions prioritized by survey participants related to runoff quantity and timing (Q2.1, 2.6), it has a distinct focus on understanding water storage and cycling at the landscape scale, including the role of groundwater (Q2.9) as well as snow, glacier and permafrost dynamics (i.e., cryosphere processes; Q2.3, 2.8).

*F3: What are the main barriers and challenges related to predicting climate change impacts and feedbacks along the land–ocean continuum?*

The greatest hindrance to understanding, and therefore predicting future change, along the land–ocean continuum is closely linked to the knowledge gaps outlined in the previous two research needs, along with those presented in the ‘Biodiversity and food web’ thematic cluster. Without a robust understanding of cross-ecosystem linkages and the key drivers and controls for these linkages, it is not possible to predict how future changes in climate or land-cover are likely to exert impacts at the landscape scale. Furthermore, even for data-rich sites, there are large discrepancies in the models currently used to describe processes in each compartment along this continuum, with large differences in drivers and parameters included, as well as differences in spatial and temporal scales and resolution. This makes is difficult to link these ‘compartments’ in order to take integrated modeling approaches to predict potential future cascading impacts across ecosystem boundaries at meaningful spatiotemporal scales.

Survey participants highlighted the need for more integrated approaches, including modeling tools, for studying processes that capture dynamic interactions along the land–ocean continuum (Q2.2, 2.4, 2.5).

*F4: How does rapid ongoing ocean change impact ecosystems and societies on coast and land?*

There is an obvious tendency to look at the catchment to coast continuum as a progressive downstream set of processes that essentially “go with the flow.” This view often sets a hierarchy where catchment change dominates the discussion and/or study design. However, rapid change is also taking place in the marine environment and it is pertinent to consider the potential effect of these. Temperature, salinity, pH, and nutrient concentrations are known to be changing in Arctic marine waters (AMAP 2021). These in turn will impart ecosystem change to both pelagic and benthic systems driving community change (CAFF 2013). This brings into question how far into estuaries such changes may take place and what impact this may have on biogeochemical cycling. For example, it could be argued that changes in coastal waters could lead to changes in standing stocks of benthic filter feeding mollusc beds in estuaries. Such changes could have profound effects on processing of both particulate and dissolved organic matter (including organic matter from land) with implications for coastal carbon cycling and balance.

Several studies have also documented upstream transport of marine-derived nutrients and carbon into freshwater and terrestrial environments via migratory fish and seabirds. For example, in North America, spawning salmon are known to be large sources of marine-derived nutrients to upstream systems, with evidence of increased terrestrial and freshwater productivity and biodiversity in salmon-influenced systems (Bartz and Naiman 2005). In the Norwegian High North, the rapid increase in invasive pink salmon (which, unlike the native Atlantic salmon, die upon spawning in freshwater systems) is also expected to alter impacted freshwater and terrestrial ecosystems, especially since many impacted systems are located in nutrient-poor tundra regions (Dunlop et al. 2021).

### Ecosystems and society: Climate adaptation

In the three northernmost counties on the Norwegian mainland, the higher precipitation, floods and surface water, and snow avalanches, rockfalls and mudflows, as well as storm surges and sea level rise are among the climate risks that are considered as the most pressing challenges in the coming years (Norwegian Centre for Climate Services 2021). In inland and mountain areas, more snowfall and mild periods in winter (i.e., zero-crossing) are also expected to increase in the next decades causing adaptation challenges for the Sámi reindeer herding community, and disrupting access to local communities (Dyrrdal et al. 2020). Ocean warming and acidification are also a looming threat to commercial fishing and aquaculture in coastal communities (Barange et al. 2018; Hänsel et al. 2020). In the High Arctic, such as the Svalbard Archipelago, people are particularly conscious about the impacts of climate change given the increase in winter temperature of 2–3 degrees combined with rainfall events, permafrost thaw affecting housing, buildings and roads, and abnormal weather events triggering avalanches, rockfalls or mudflows (Hovelsrud et al. 2020; Timlin et al. 2022). Despite these climate risks, and in contrast to the Svalbard archipelago, the municipalities in the Norwegian High North are responding slowly, at least if measured by applications to the climate adaptation fund (*KBNN* 2021). Municipalities tend to interpret adaptation differently, with a strong bias toward mitigating and preparing for physical climate risks and damage to building, infrastructure, and transport (Selseng et al. 2021). Climate adaptation is also primarily incremental, short- and medium term and targeted toward sectors, rather than taking a system approach and a long-term perspective for transformative adaptation (*KBNN* 2021).

The elevated climate risks combined with the slow response of local communities may appear as a paradox. The reasons for the lack of climate adaptation planning and action could be many, including lack of usable or actionable climate information (i.e., climate services), lack of awareness, low capacity or even climate skepticism (as exemplified by one of the survey participants who suggested Q3.13: How to depoliticize the issue of climate change?). The question is highly relevant, but it is not explicitly coupled to cross-ecosystem and socioecological impacts of climate change. Similarly, three of the questions prioritized risk-hazard approaches (Q3.3–3.5), emphasizing the need to assess vulnerability and specific risks to societal sectors. One question was related to how traditional Sámi reindeer pastoralism will transform to become more dependent on artificial feeding and motorized transport (Q3.11), and another focused on coastal erosion (Q3.12). All these questions are relevant to climate adaptation, but in the survey, we asked about relevance for adaptation to cross-ecosystem impacts and its underlying socioecological dynamics. The research needs for this topic, highlighted below, integrate themes from participants’ survey responses while focusing on cross-ecosystem impacts in relation to climate adaptation.

*C1: What are the impacts of climate mitigation measures (e.g., connected to renewable power, infrastructures) on socioecological systems, and how could such actions be planned to co-benefit biological diversity, climate resilience and local and Indigenous opportunities to adapt to climate change?*

In relation to the question posed above, many of these topics consider climate adaptation in the context of increased use of land, rivers, and seas for other purposes, especially relating to the green transition (e.g., hydropower development, battery production, windmills, and associated infrastructures such as roads and cables etc.) (Q3.1, 3.2, 3.7, 3.8, and 3.14). Nature-based solutions are actions to protect, sustainably manage, and restore natural and modified ecosystems that address societal challenges effectively and adaptively, simultaneously benefiting people and nature (Cohen-Shacham et al. 2016). Whereas these questions could be included as a part of co-producing nature-based solutions, they are broader in scope in terms of understanding the tradeoffs between the need for climate mitigation, adaptation and other needs associated with the UN Sustainable Development Goals. The concern about the increase in green energy development was expressed by many in the survey and therefore justifies a question entirely devoted to this topic. This is also a research question of international importance. This question is related to transformative adaptation that moves beyond reactive or incremental adaptation by embracing system-wide change across more than one system; that plans for a long-term horizon; that includes a desired sustainable future by focusing on root causes for sustainable development; and that critically scrutinizes the effectiveness of current practices of climate adaptation (or lack thereof) by examining social injustices and power imbalances (Lonsdale et al. 2015; Boon et al. 2021; Filho et al. 2022).

*C2: How can researchers downscale climate projections and mobilize climate data for the assessment of cross-ecosystem impacts in ways that are meaningful and scale appropriate to enable locally relevant adaptation actions?*

Climate modeling and forecasting are generally global in scale and require downscaling to ensure relevance to local landscapes and communities and to inform adaptation actions. Translating global-scale models to be relevant at local scales (whether through the use of regional models or statistical functions) is a necessary step to control for uncertainty of predictions at the scales at which ecosystem sampling takes place (Pielke Sr and Wilby 2012). Furthermore, with the growing availability of high-resolution remote-sensing data, there are increasing opportunities to relate climate models to high-quality land and water surface measurements, and to validate climate models with remotely sensed data at regional scales (Wang 2023). One way of translating all the stated research questions from the survey into a topic of relevance to cross-ecosystem and socioecological impact is by developing appropriate climate services. Climate services offer scientifically credible information that directly respond to user needs for adaptation and decision-making (Boon et al. 2021). The Norwegian Climate Service already provides usable knowledge for understanding physical climate risks on local scale, but for understanding the socioecological consequences and necessary actions, there is a need for targeted information that is built through the appropriate two-way engagement between the users and providers. Information regarding socioecological climate risks is often not comprehensive and can be difficult to access. However, decision-makers at the local scale can still benefit from comparisons with information from other risk assessments at local scales. Data needed include scenarios about impacts to different economic activities that are important at the local level (i.e., agriculture, fisheries, etc.), assessment of risks to transportation systems, ways to measure place attachment and quality of life under climate change, and assessments of the impact of climate stress on well-being. One of our priorities is therefore to communicate consequences that link climate, ecosystems, and society in a way that is more accessible to users and local decision-makers. Such climate services could also increase community engagement for example by involving schools, as suggested in Q3.16. Many of the data required for holistic socioecological adaptation could be co-produced with schools and in other areas of the community. This would in turn contribute to long-term legitimacy of the data mobilization and resulting adaptation actions by placing agency in the hands of the younger generations and at the local level.

*C3: What are the socioecological impacts of extreme climate events and what are the potential transformation pathways (including immediate actions) necessary to cope with and adapt to these events?*

There is a need for transformative change to respond to the anticipated increase in frequency and magnitude of extreme events and their impacts on socioecological systems. The Sixth IPCC assessment provided a strong message that the world must prepare for the increase in frequency and intensity of extreme events that affect food and water security, livelihoods, health, buildings and infrastructure. None of the questions mentioned extremes explicitly, although a few mentioned the societal impacts of climate risks (Q3.3–3.5). To develop transformative pathways for adapting to climate risks, including novel ones, there is a need to build on plausible scenarios of change in frequency and intensity of extreme events, which were mentioned in Q3.10.

### Ecosystems and society: Management

Effective management strategies and adaptive measures by communities and society are pivotal to address the impacts of climate change on ecosystems and societies. Climate change involves cascading effects and can be coupled with feedbacks that flow across ecosystems and thus society. These effects and feedbacks can have unpredictable outcomes and present one of the main challenges for management strategies. Management responses to meet the challenges of climate change, human development, and environmental stressors can also affect society in general as well as local communities (Q4.6, 4.7 and 4.10). Given the linkage between social and ecological systems, it is thus crucial for future management of northern ecosystems to take these aspects into consideration.

*M1: What are the shortcomings in existing management frameworks and practices to meet future challenges related to cross-ecosystems impacts in a more holistic and adept way?*

Survey responses recognized the importance of assessing existing monitoring data, filling data gaps, and establishing long-term monitoring to build management actions on a foundation of scientific knowledge of climate change impacts on ecosystem structure and function (Q4.3, 4.4, 4.5, 4.8, 4.11, 4.12, and 4.13). Both survey responses and expert discussions highlighted the importance of this knowledge base to inform policy and management approaches and actions but recognized that monitoring to support this knowledge base must be routine and must occur over a minimum of 10 years to detect long-term impacts of climate change. Management decisions must be based on an understanding of the current state, historical changes, and projected shifts to ecosystems, and should consider ecosystem components that contribute to ecosystem services (Q4.2). In particular, impacts of climate change on businesses and industries that rely on the land (e.g., agriculture, fishing, fish farming, reindeer husbandry) were identified as a key concern for management (Q4.1, 4.7, 4.10) and highlighted the importance of working with the Sami People to co-develop management approaches (Q4.11).

Management initiatives need to meet these challenges in legitimate and adept ways which integrate the relationships between social and ecological systems and climate change as well as potential changes of legal frameworks and policies at different levels into consideration. A better understanding of how management frameworks currently address the impacts of climate change and local developments across ecosystems is important as a basis for developing a more holistic management approach and contributing to management-relevant knowledge to meet challenges of climate change adaptation across ecosystems (Q4.8). Therefore, insights into how knowledge is currently generated and used in the selected management frameworks, what assessments are made and how participation is taken into account should be a priority on the research agenda to cope with the future challenges. Climate change in addition to increasing human development may induce complex dynamic alterations of ecosystems in the Arctic. So far monitoring of ecosystems has been mostly done without integrating cross-ecosystems links and the effects of climate change, which provides limited knowledge for ecosystem-based management (Andersson et al. 2015). Future management framework needs to adopt a framework that structures and integrates the relationships between these systems. A better understanding of the linkages between these systems and an increased communication of human–environment interactions coupled with adapted policy developments could improve our capacities to meet future challenges.

*M2: What kind of early warning systems do we need and how can we manage emergent (e.g., generalist mesopredators) and invasive species (e.g., pink salmon, mosquitoes), pathogens (e.g., virus) and pests (e.g., moth outbreaks) and humans health, safety, and well-being (e.g., Opmu, drinking water quality, transport)?*

Globally, the introduction of non-native species is considered a major threat to biodiversity and ecosystem services. The low diversity of native species in the Arctic and the increased human activities in addition to a warming climate is putting the Arctic in a particularly vulnerable position to threats. Invasive species are introduced through human activity and by spreading in a new habitat, they threaten native species and the functioning of ecosystems (Pejchar and Mooney 2009). In Norway, the invasive pink salmon has been observed in rivers along the coast in increasing numbers since 1960. The spreading of the pink salmon can impact the ecosystem of Norwegian rivers in multiple ways through new diseases, changes in nutrient flux (high number of dead salmon in the rivers after spawning) or by competing with native species for habitat and food. The spread of disease can have direct impact on salmon farming in Norwegian fjords and the influx of a large number of dead and dying fish as a new food source for terrestrial animals can have unpredictable consequences on the ecosystem (Mo et al. 2018; Sandlund et al. 2019). Human activities and climate change are impacting all ecosystems around the world and invasive species, pathogens, pests are threatening the ecosystems and the underlying socioecological dynamics and feedbacks. It is crucial for the protection of the Arctic and sub-Arctic ecosystems and for human well-being to develop better response programs by undertaking prevention and early detection of possible threats. In the case of invasive species in the Arctic and sub-Arctic, there exist strategies and action plans with a framework and guidelines for priority actions (CAFF and PAME 2017). Participants also mentioned the need to bring in local—and Indigenous experiences to understand climate-related events. One example is *Opmu* in Sámi language which refers to a large mud hole covered by green mosses. Sámi reindeer herders have observed that these miry holes are open during the winter to a greater extent today compared to earlier years according to one of the participants and thus poses a danger to reindeer and reindeer herders as these are difficult to detect in winter. Here, we emphasize the need for research to become better at predicting and forecasting changes at different scales by increasing collaboration and knowledge sharing between resource users, researchers, and decision-makers, facilitating improvements to early warnings and a faster response to potential threats.

*M3: How can co-production of knowledge with Sami and local communities contribute to developing locally relevant nature-based solutions and improving ecosystem management?*

Questions to both the management and the climate adaptation group emphasized the need for consideration of Sami Traditional Knowledge of nature together with Western science to create a more holistic knowledge base for nature management, climate policy, and economic development (Q3.9, Q4.11). Indigenous Knowledge and Western science represent different knowledge systems, and both can contribute to our understanding of the impacts of climate change (Alexander et al. 2011; Mistry and Berardi 2016; Tengö et al. 2017). However, it is important that managers and policy makers recognize the complementarity of these two ways of knowing, and do not seek to assimilate or institutionalize Indigenous Knowledge within Western knowledge systems (Mistry and Berardi 2016). Rather, there is opportunity to acknowledge the value of Indigenous observations and methodologies and to braid Indigenous Knowledge with Western science in a way that maintains the separate and unique identities of each knowledge system while joining together what is common between them (Tengö et al. 2017). Through knowledge exchange and equal valuation of knowledge systems, a more holistic knowledge base that builds on past observations and experiences can be developed (Tengö et al. 2017).

Many of the questions for the management and climate adaptation groups recognized the protection of nature as necessary for adapting to climate change (Q3.1, 3.2, 3.6, and 3.11) and emphasized the need for management approaches to consider preservation of ecosystem services and sustainable use of resources (Q4.1, 4.2, 4.6, 4.7, 4.10). In the panels we discussed that many of these research needs could be coupled to nature-based solutions, for example by ecosystem-based adaptation that recognizes the capacity of ecosystems and diverse natural pastures to buffer Sámi communities against the adverse impacts of climate change (Hausner et al. 2020). Nature-based solutions that are co-produced are more likely to be meaningful for local adaptation to climate risks and also have a higher likelihood of engaging Indigenous- and local communities as part of management actions. This co-production of management approaches and activities is key if adaptation actions are to be locally relevant, realistic, and successful (Heino et al. 2020).

*M4: How can researchers and management agencies communicate the results of monitoring and assessments in ways that are meaningful to stakeholders, will inform policy decisions, and will engage communities?*

Panel discussions highlighted the important role of communication in ensuring all relevant stakeholders (including Indigenous and local communities, researchers, managers, and policy makers) are engaged in the management process. Heino et al. (2020) described the importance of communication between stakeholders to ensure management actions and policies to preserve Arctic ecosystems consider environmental and socioeconomic needs. Indigenous and local communities should be engaged in the co-development and implementation of monitoring and assessment activities to ensure they address locally relevant questions and concerns. The results of these activities should then be communicated in a way that is meaningful to all local stakeholders. Further, communication to policy makers must be relevant to policy questions and highlight actions that can be taken to support preservation of ecosystems and the services they provide. Increased communication and interaction between stakeholders at all levels is necessary to ensure management and policy are supported by a strong scientific foundation and to facilitate community engagement in monitoring and management actions.

## Concluding remarks

The 15 research needs we identified in this horizon scan cover several multidisciplinary themes in relation to climate change impacts on cross-ecosystem linkages and the underlying socioecological dynamics and feedbacks in the Norwegian High North. These research needs are relevant and urgent to address for a region that is changing rapidly. Prior studies using different methods have also identified emerging research needs and knowledge gaps in the Arctic and sub-Arctic (National Research Council 2014; Ancin Murguzur and Hausner 2020). Eight years ago, the National Research Council (2014) highlighted the emerging research questions across different fields of Arctic research in relation to the changes impacting physical, biological, and social systems. They drew an overview of the main gaps in Arctic research, highlighting the need to improve cooperation among researchers as well as between researchers and decision-makers. The research questions identified in their study remain highly relevant, but our distinct goal was to highlight the knowledge gaps that relate to cross-ecosystem impacts as well as to understanding and responding to rapid changes in social-ecological systems in the Norwegian High North. In addition, we would like to emphasize the need to integrate not only decision-makers in the process but also a broad range of stakeholders that can provide diverse perspectives and contribute valuable knowledge and lead to an improved and more holistic system understanding. We recommend that our research priorities be reflected in the programs of research funders from the public sector and other potential funders to ensure research is oriented to understanding and tackling urgent societal challenges. As highlighted in previous studies, our findings illustrate the lack of inter and multidisciplinary research in Arctic research and the long-lasting dominance of Arctic and sub-Arctic research toward natural sciences versus the urgent need to integrate social sciences for climate adaptation (Ancin Murguzur and Hausner 2020). Nevertheless, climate-related challenges as well as research are constantly evolving, therefore the horizon scanning process we went through here should be reviewed frequently to consider whether the gaps have been filled and other research questions have become more important.

There have been global horizon scans where emerging global issues in conservation or in climate science were identified (e.g., Sutherland et al. 2020; Martin et al. 2021) but our panel of experts focused specifically on research needs for the Norwegian High North. Region-specific analyses of knowledge gaps, targeted to climate-related challenges are needed, especially in regions predicted to experience the more extreme impacts of climatic changes, as in the Norwegian High North. Prioritizing research needs in response to regional and local scales provides more insight into ecosystem-based adaptation and management strategies, benefiting both ecosystems and the communities who live with and depend on them. We suggest that our method offers a useful example to others of how such regional-level prioritization may be achieved.

Horizon scanning is valuable both for its final product and for its process of creating transdisciplinary cooperation and knowledge exchange. It also revealed the particular challenges associated with the complexity of the processes involved in understanding cross-ecosystems linkages represented by research questions identified in our study. However, we also noticed uneven participation which may limit the representativeness of the results presented here. Specifically, participants coming from academia tended to be more active and engaged than non-academics in refining the questions; additionally, academics tended to focus more on long-term and system-oriented research versus the specific knowledge needs requested by specific sectors. Such challenges are probably not limited to our experience, other studies of participatory scenario-planning have reflected on the challenges of making a process simultaneously salient to different knowledge-holders (Waylen et al. 2015; Wheeler et al. 2020). For future horizon scans we must consider when and how best to involve and empower local representatives in all stages of the process, perhaps focusing on deliberations subsequent to a horizon scan report, using other tools and participatory approaches to elicit deliberation and implications and actions relating to climate change risk reduction and adaptation (see, e.g., Hill et al. 2020). Developing and reflecting on methods that value and engage the perspectives of different knowledge-holders is an important challenge for us and all transdisciplinary engagement processes.

Our research needs reflect but also go beyond the issues highlighted by previous individual studies. Here, we emphasize the need to understand climate change impacts that go beyond narrow studies (or management approaches) focusing on single ecosystems (e.g., terrestrial, freshwater, marine) toward a ‘catchment to coast’ understanding including the underlying socioecological dynamics and feedbacks linked to it. Despite the strong interest of research toward ecosystems modifications with warming climate in the Arctic, few studies address the potential societal implications of climate change (Ancin Murguzur and Hausner 2020). Both the global horizon scans and our horizon scan consistently suggest that climate change by affecting the environment could drastically affect the social and economic systems linked to it. Global reviews of indirect drivers of change also highlight the importance of reciprocal influences from society onto nature (e.g., Díaz et al. 2015) It is therefore urgent to identify the society and ecosystems as having multiple interlinked interactions. The results of this horizon scan offer important transdisciplinary insights for research and practice. An urgent challenge is now to develop and tackle the priority research needs outlined above, in order to mitigate the effects of climate change in the Norwegian High North.

## Supplementary Information

Below is the link to the electronic supplementary material.

Supplementary file1 (PDF 152 KB)
