# Supplementary material for: Fifteen research needs for understanding climate change impacts on ecosystems and society in the Norwegian High North
Source: Ambio. 2023 Jun 7;52(10):1575–91. doi: 10.1007/s13280-023-01882-9 (PMC10460749; doi:10.1007/s13280-023-01882-9)
Supplement: Supplementary file 1 — Supplementary file1 (PDF 152 KB) [file 13280_2023_1882_MOESM1_ESM.pdf]

Ambio

Supplementary Information
This supplementary information has not been peer reviewed.

Title: **Fifteen research needs for understanding climate change impacts on ecosystems and society in the Norwegian High North**

Authors: Zina Kebir, Catherine Chambers, André Frainier, Vera Hausner, Ann Eileen Lennert, Jennifer Lento, Amanda Poste, Virve Ravolainen, Angelika H. H. Renner, David N. Thomas, Kerry Waylen

Appendix S1: Survey Results

Biodiversity and Food Web

1.1 How resilient are ecosystems to climate change and what factors may cause a shift to alternative stable states?

1.2 What are the main changing environmental factors and their relationships to ecosystem functioning?

1.3 The significance of climate change for migratory birds, changes in migration time, breeding time. Possible changes in food supply due to increase in temp. Applies to both seabirds and species on land.

1.4 What consequences does it have for the local species (plants, birds and animals) that the plateau / tundra grows back from shrub vegetation? That the tree line is constantly creeping higher up. Penetration of new species, loss of biodiversity, changes in ecosystem functions...

1.5 What do the temperature changes / increases have to say for the native local species. Plant and animal species. Will this affect the locals' opportunities for hunting, berry picking and fishing? Will cloudberries, for example, eventually disappear from our bogs because the permafrost thaws?

1.6 How can food webs be compared across ecosystems?

1.7 Will reindeer continue to be able to graze in Finnmark in the future? If not, what will happen to the landscape and environment?

1.8 What is the impact of ocean acidification on marine resources?

1.9 Use of eDNA to monitor diversity at all levels, including the prokaryotic diversity that is often central to metabolism.

1.10 (EXTRA) How can the negative impact from farming and other influencing factors on salmon be better documented, and how can the knowledge base for the management of coastal fishing, including Sea Sámi fishing, be strengthened?

Climate-Land-Water:Fluxes

2.1 Climate effect on runoff: How does changes in increased winter temperatures (which again affect frozen ground, snow and rain) affect quality of runoff from land. Quality in the broadest sense (but particularly the load of nutrients and pollutants).

2.2 It may be obvious with the name of the project, but central is the integration of different systems; atmosphere, land, fresh water, coast, where water and hydrology are the integral element. There should be an increased focus on freshwater as an element to capture changes in terrestrial systems, and which bring the changes on to marine recipients. Carbon, nitrogen, phosphorus, silicon and iron are key elements for production and should be followed in an integrated manner.

2.3 What will be the ecological consequences of permafrost thaw?

2.4 Improved models that look at the land-ocean continuum as a coupled system

2.5 How will future changes (climate, land cover, insect attacks) affect the terrestrial and aquatic carbon cycle? Will it lead to positive feedback on climate?

2.6 How will climate change affect river flows, flood risk (surface water in built-up areas on permafrost and ice-covered ground) and sediment transport? What impact will it have on human movement and nature interactions?

2.7 Research on feedback mechanisms, primarily how the mobilization of organic carbon, together with N and P, stimulates the production of CO2 and CH4. Should also be linked to eDNA and RNAseq to look at microbial gene activity

2.8 Avalanches - change in snow types and amounts of snow as a result of climate change? Will climate change mean that classic avalanche protection against slab avalanches / loose snow avalanches is not sufficient against changing types of avalanches? That is, will the rock falls get bigger, or will the snow be wetter and heavier, towards slippery slides?

2.9 Groundwater-related ecosystems in the north.

2.10 (EXTRA) How can grazing on natural pastures / outfield pastures stimulate carbon storage in pasture soils? how can this knowledge be integrated into climate accounting to balance the one-sided negative impression of emissions from grazing animals as a climate problem

Ecosystems and Society: Climate adaptation

3.1 How to ensure better protection of nature? Increasingly large areas are being demolished with cabins, roads, wind power, etc, and the natural areas are both deteriorating and fragmented. This in turn makes nature more vulnerable to climate change.

3.2 Explore and highlight harmful consequences for nature of climate measures such as a. development of wind power plants and mining activities that will support the so-called green shift, which is in danger of becoming the gray shift when nature destruction is not made visible and emphasized in decision-making processes, and including strengthening knowledge about what researchers at NINA call nature cure - synergy between nature conservation and climate measures - as a supplement to the authorities' climate cure.

3.3 How will climate change affect human societies in the north? What impact will it have on infrastructure, industry and security?

3.4 How to adapt to climate change? Increased research on preventive measures in a cost-benefit perspective in various areas. Most people want to adapt, but there is uncertainty about how this should be done and what effect it actually has.

3.5 Vulnerability (ROS) for impacts of climate change - on society, cultural heritage / cultural practice, settlement in the north.

3.6 How to safeguard our northern resources in the most ecologically sustainable way possible? Intensive conventional agriculture depends on phosphate, which is a very limited resource in the long run. The war in Ukraine also illustrates that it can be risky to be too dependent on imported food. Strengthened knowledge of our northern terrestrial, limnic and marine ecosystems and agriculture is therefore absolutely necessary to secure our livelihood in the north with nature preserved.

3.7 How to reduce energy consumption instead of facilitating increased energy production. Whatever form of energy we choose, it has negative consequences for nature and society in the short and especially long term. Therefore, our energy consumption must be greatly reduced - and a lot of energy is wasted. This also includes completely unnecessary transport of goods around the world to

find cheaper processing sites, where both transport and polluting power contribute to very negative emissions to nature.

3.8 Explore, highlight and strengthen the knowledge base to see the sustainability goals and identify synergies between goals related to climate and nature, and goals for economics, socio-economic and social conditions and culture, and strengthen the overall knowledge base for political trade-offs between goals to promote the green shift and other societal goals.

3.9 Explore and highlight the importance of reindeer husbandry, sea and river fishing and other Sámi outdoor activities as a basis for Sámi culture and as a basis for our common nature to be protected from destructive degradation, including ensuring stronger integration of Sámi traditional knowledge of nature with scientific knowledge in a comprehensive knowledge base for nature management, climate policy and economic development.

3.10 Identify plausible trajectories of environmental and societal changes in the near future compatible with scenarios for changes in the distant future (i.e. to avoid jeopardizing future options).

3.11 The significance of climate change for traditional Sámi domestic reindeer husbandry in its original, nomadic form. Without support feeding and transport of reindeer from winter to summer grazing with eg trailer. Mild winters have already shown what consequences it may have for reindeer husbandry in the future. Is it possible with adjustments? It is precisely the natural way in which reindeer live that makes reindeer meat as exclusive as food, for example. If the animals are fed, the taste and meat quality change.

3.12 Coastal erosion - where (geographical and or type of bedrock / geomorphology), how fast does it happen, how can coastal erosion against buildings / infrastructure be prevented / reduced, what measures are good measures to "save" buildings and infrastructure that are exposed to coastal erosion? What is a safe building site against coastal erosion - can one calculate how far inland the erosion will take place and how fast it will erode?

3.13 How to depoliticize the issue of climate change?

3.14 (EXTRA) How can we make visible the consequences for nature and resource use of increased pressure to develop industry for data storage and battery production, and how is the room for maneuver for Norwegian environmental management affected by the fact that foreign ownership interests are strong in these areas?

3.15 (EXTRA) How can we make visible the potential for increased use of geothermal energy in Norway, for homes and industry, and highlight how the consequences for nature will be less than with e.g. wind power, and develop the socio-economic cost assessment to also include reduced damage to nature?

3.16 (EXTRA) How to integrate this knowledge in a school / teaching context? By increasing the knowledge of future generations, ie children and young people, it will be possible to ensure that in the long run greater and better consideration is given to climate adaptations in community planning.

<u>Ecosystems and Society: Management</u>

4.1 Understand the synergistic effects between climate change and other processes that affect ecosystems, including plans for new business activities and land use, and communicate the understanding through assessments that address relevant decision-makers and the general public.

4.2 What are the impacts of climate change on ecosystem services derived from local, regional and above regional biodiversity?

4.3 Filling up the data gaps by compiling older data where it hasn't been done yet.

4.4 Monitoring of changes in relation to baseline data over a minimum of a ten-year period.

4.5 Establish integrated monitoring systems that make it possible to identify pathways for adaptation across different socio-ecosystems and to distinguish the effects of management versus other changes (eg climate).

4.6 Impact on existing drinking water sources and the need for development / securing of new sources without affecting existing ecosystems and biological diversity.

4.7 The sea - how climate change affects temperature, local climate and ecosystem in the fjords. Through farming of salmon along the Norwegian coast, large amounts of food are produced, and this creates large ripple effects. How will salmon farming (other species as well) and the overall fishing industry be affected by climate change?

4.8 How to develop better methodology for action planning based on the knowledge from comprehensive assessments ?

4.9 Identify possible management policies and how they can be assessed robustly and as quickly as possible using (quasi) experimental approaches.

4.10 Consequences for reindeer husbandry and increasingly demanding conditions in the winter grazing areas.

4.11 (EXTRA) Strengthen the knowledge base for management by combining scientific knowledge and traditional knowledge, both from Sami industries and outdoor use and from agriculture and fishing.

4.12 (EXTRA) Strengthen the knowledge base for precautionary perspectives in management, by highlighting the sum effect, cumulative effect of climate change, land encroachment and management, and identifying early warnings.

4.13 (EXTRA) Strengthen the knowledge base for management by documenting in what way untouched nature is the best climate measure

Table S1. Creation of final list of research needs from the original questions from the survey

Final research needs	Discussion Full group	Questions after group work	Original questions number
Biodiversity and Food web			
B1.If one ecosystem changes its ecosystem state (e.g., from kelp-dominated to urchin-dominated coastal zone, from tundra to birch forest ecosystem, from benthic-dominated to pelagic-dominated aquatic system or vice-versa) (1) do the other systems change, (2) how do they change, and (3) how large this change has to be in order to change the other ecosystem?	Examples added	If one ecosystem changes its ecosystem state, (1) do the other systems change, (2) how do they change, and (3) how big the change in one ecosystem has to be in order to change the other ecosystem?	Q1.1,1.2,1.6
B2.How will climate change impact ecosystems structure (e.g. biodiversity, vegetation strata), functions (flux of nutrients) and services they can provide (e.g. grazing, livestock, berries, game and fish, tourism, cultural, flood protection, aquaculture)?	Reformulated + examples added	How will climate-driven changes in the tundra ecosystem change its structure (vegetation, species composition), function (flux of nutrients), and services they provide (hunting, grazing, berry picking, fishing)?	Q1.3,1.4,1.5,1.7
B3.What is the cumulative impact of climate change and anthropogenic stressors on biological taxa that are dependent on or act as links between multiple ecosystems?	Reformulated to emphasize on the anthropogenic impacts	What is the cumulative impact of climate change on biological taxa that are dependent on multiple ecosystems? What happens to those species is important because they provide linkages across ecosystems.	Novel
B4.How do changes in frequency and magnitude of climate events (extreme events including abrupt increase/decrease in precipitation, temperature, and flood) affect food web connections across ecosystems (e.g., from sea to land, from freshwater to land, from land to sea), and which adaptation actions (immediate and long-term) are necessary to respond to these changes?	Reformulated	How do changes in frequency and magnitutde of climate events (precipitation, temperature, …) affect food web connections and the flux of resources across ecosystems (e.g., mosquitoes that emerge in bogs and are food to birds and affect reindeer and pollinate plants near and far)	Novel
	Merged with B2	What consequences does it have for the local species when the temperature changes/increases and the tundra/plateau grows back from shrub vegetation? Particularly for migrating birds, changes in species composition, ecosystem function, …	Q1.4
Climate-Land-Water: Fluxes			
F1.What are the effects of climate change (including changes in seasonality and extreme events) and land-use change on biogeochemical fluxes and water quantity and quality along the terrestrial-freshwater-marine continuum?	Reformulated	Effects of climate (terrestrial and marine) and land-use change and shifting phenology on freshwater and marine water quality?	Q2.2,2.1

F2.What are the implications of changed storage times in different water reservoirs (e.g. groundwater, surface water, snow, glaciers, fjords) within the land-ocean continuum?	Added examples	What would be the effects of changed storage times in different water reservoirs along the land-ocean continuum?	Q2.1,2.2,2.4,2.9
F3.What are the main barriers and challenges related to predicting climate change impacts and feedbacks along the land-ocean continuum?	Reformulated	What are the main barriers and challenges related to predicting climate change impacts along the land-ocean continuum?	Novel
F4.How does rapid ongoing ocean change impact ecosystems and societies on coast and land?	New (MISSING MARINE QUESTION)		
	Merged in B4	What will happen with natural hazards (e.g. floods, avalanches, permafrost destabilization), and what are the consequences for ecosystems and humans?	Q2.3,2.8,2.1,2.6
	Merged in F1	How will cc + land-use change impact contaminants/pollutants along the continuum?	Q2.1
Ecosystems and society: Climate adaptation			
C1.What are the impacts of climate mitigation measures (e.g. connected to renewable power, infrastructures) on socioecological systems, and how could such actions be planned to co-benefit biological diversity, climate resilience and local and Indigenous opportunities to adapt to climate change?	Added examples	What are the impacts of climate mitigation measures on socioecological systems, and how could such actions be planned to co-benefit biological diversity, climate resilience and local and Indigenous opportunities to adapt to climate change?	Q3.1,3.2,3.5,3.8
C2.How can researchers downscale climate projections and mobilize climate data for the assessment of cross-ecosystem impacts in ways that are meaningful and scale appropriate to enable locally relevant adaptation actions?	Reformulated	How can we downscale and communicate cross-ecosystem impact science to enable or provide for locally relevant adaption actions (in response to for example development, extreme events, changing global markets, etc)?	Q3.16
C3.What are the socioecological impacts of extreme climate events and what are the potential transformation pathways (including immediate actions) necessary to cope with and adapt to these events?	Reformulated	What are the potential pathways including the immediate actions necessary to transform socio-ecological systems to cope with extreme events and adapt to climate change?	Q3.10,3.11
Ecosystems and society: Management			
M1.What are the shortcomings in existing management frameworks and practices to meet future challenges related to cross-ecosystems impacts in a more holistic and adept way?	Reformulated	What are the current challenges in management frameworks and practices to meet future challenges of climate change impacts and environmental change in more holistic and adept ways?	Q4.8, Q4.3

M2.What kind of early warning systems do we need and how can we manage emergent (e.g. generalist mesopredators) and invasive species (e.g. pink salmon, mosquitoes), pathogens (e.g. virus) and pests (e.g. moth outbreaks) and humans health, safety and well-being (e.g. Opmo, drinking water quality, transport)?	Reformulated	What knowledge and methods are needed to manage the invasion of pink salmon and reduce the consequences on the wild Atlantic salmon and ecosystem services connected to salmon fishing in rivers as well as in the sea?	Q4.11,4.12,4. 8,4.9, 4.2, 4.7
M3.How can coproduction of knowledge with Sami and local communities contribute to developing locally relevant nature-based solutions and improving ecosystem management?	Reformulated and moved durining editing step	How can we combine Sami and local ecological knowledge and scientific knowledge to identify and achieve locally beneficial nature-based solutions?	Q3.8,3.9,3.11
M4.How can researchers and management agencies communicate the results of monitoring and assessments in ways that are meaningful to stakeholders, will inform policy decisions, and will engage communities?	Originally merged in C2; Reformulated (during editing step)	How can researchers and management agencies communicate the results of monitoring and assessments in ways that are meaningful to stakeholders and that will inform policy decisions?	Q4.1
	Merged in B2	What are the impacts of climate change on ecosystem services derived from local, regional and above regional biodiversity, with emphasis on the consequences on ecosystem services related to salmon farming and on harvesting of game, fish, berries and plants?	Q4.7,4.2,4.6
	Merged in C3	How can we braid western science and indigenous methods to monitor changes in relation to baseline data (established in part by compiling other data to fill data gaps) over a long time period, included integrated holistic monitoring and impact assessments of whole ecosystems and across ecosystems (terrestrial, freshwater)?	Q4.3,4.4,4.5

Appendix S2: GROUP WORK organization:

Equipment per group:

- One computer (Zoom, Word and Online Microsoft FORM open)
- Printed full list of questions for each participant
- Paper, pen, pencils

	Time	Activity
1st Round	5-10 min	***Individual input***<br>(Each participant writes individually on paper)<br>- Prioritize one question/topic on the list corresponding to the group's theme. You can also merge questions/topics.<br>- If necessary, clarify or reformulate the topic/question<br>- Prepare to explain with your words the reason for prioritizing this question/topic based on novelty and/or societal relevance.
2nd Round	20-30min	***Synthesizing and clarification***<br>Each participant takes turn to present the question and explain why this question is important (ca. 5min per participant)<br>The leader writes down the questions (WORD DOCUMENT)
3rd Round	5-10min	***Group Discussion***<br>Clarification of research questions and synthesis<br>The leader takes notes (WORD DOCUMENT)
BREAK	10min	
4th Round	5-10 min	***Individual input***<br>(Each participant writes individually on paper)<br>- Pick up one question in the list corresponding to your theme that hasn't been chosen before **OR** you can suggest a new question that you think is missing in the theme<br>- Clarify and reformulate if necessary<br>- Prepare to explain with your words the reason for prioritizing this question/topic based on novelty and/or societal relevance.<br>-
5th Round	20-30 min	***Synthesizing and clarification***<br>Each participant takes turn to present the question and explain (ca. 5min per participant)<br>The leader writes down the questions on the computer (WORD DOCUMENT)
6th Round	10-20min	***Group Discussion + Vote***<br>Clarification of research questions and synthesis<br>Vote by raising hands on 3 to 5 topics that should be retained<br>Leader writes down the final list of questions for the group in the online form (MICROSOFT FORM)
